# Activity-Mediated AMPA Receptor Remodeling, Driven by Alternative Splicing in the Ligand-Binding Domain

**DOI:** 10.1016/j.neuron.2012.08.010

**Published:** 2012-11-08

**Authors:** Andrew C. Penn, Ales Balik, Christian Wozny, Ondrej Cais, Ingo H. Greger

**Affiliations:** 1 Neurobiology Division, MRC Laboratory of Molecular Biology, Hills Road, Cambridge CB2 0QH, UK

## Abstract

The AMPA-type glutamate receptor (AMPAR) subunit composition shapes synaptic transmission and varies throughout development and in response to different input patterns. Here, we show that chronic activity deprivation gives rise to synaptic AMPAR responses with enhanced fidelity. Extrasynaptic AMPARs exhibited changes in kinetics and pharmacology associated with splicing of the alternative flip/flop exons. AMPAR mRNA indeed exhibited reprogramming of the flip/flop exons for GluA1 and GluA2 subunits in response to activity, selectively in the CA1 subfield. However, the functional changes did not directly correlate with the mRNA expression profiles but result from altered assembly of GluA1/GluA2 subunit splice variants, uncovering an additional regulatory role for flip/flop splicing in excitatory signaling. Our results suggest that activity-dependent AMPAR remodeling underlies changes in short-term synaptic plasticity and provides a mechanism for neuronal homeostasis.

## Highlights

► Homeostatic mechanism intrinsic to the AMPA receptor ► Circuitry-selective activity-driven alternative AMPA receptor splicing ► Neuronal homeostasis via AMPA receptor short-term plasticity in CA1 ► Functional capacity for flip/flop splicing in excitatory signaling

## Teaser

Penn et al. show that AMPA receptor kinetics are adjusted homeostatically in response to chronic activity changes. This involves changes in subunit assembly and is driven by alternative flip/flop splicing. This mechanism is subfield specific and is restricted to CA1 hippocampus.

## Introduction

AMPA-type glutamate receptors (AMPARs) initiate postsynaptic signaling at excitatory synapses (Traynelis et al., 2010; Trussell, 1999). Receptor desensitization can shape synaptic transmission and in turn information processing (Chen et al., 2002; Koike-Tani et al., 2008; Rozov et al., 2001; Xu-Friedman and Regehr, 2003) as a function of the cleft glutamate transient (Cathala et al., 2005; Jonas, 2000; Xu-Friedman and Regehr, 2003). AMPAR kinetics are tuned by the composition and alternative RNA processing of the four core subunits (GluA1–GluA4) (Geiger et al., 1995; Jonas, 2000) and by auxiliary factors (Guzman and Jonas, 2010; Jackson and Nicoll, 2011). Neurons express a variety of functionally distinct AMPARs, which can be recruited selectively in response to different input patterns (Liu and Cull-Candy, 2000) and be targeted to specific dendritic subdomains (Bagal et al., 2005; Gardner et al., 1999; Tóth and McBain, 1998). However, whether assembly into distinct heteromers is modulated by activity is not known (Pozo and Goda, 2010; Turrigiano, 2008). Activity-driven remodeling of kinetically distinct receptors would permit adaptive responses to changing input patterns.

The ion channel and ligand-binding domain (LBD) of the receptor feature regulatory elements at subunit interfaces introduced by alternative RNA processing (Seeburg, 1996). Q/R editing at the A2 channel pore controls Ca^2+^ flux and receptor tetramerization (Greger et al., 2003; Isaac et al., 2007), whereas the R/G editing and alternative splicing within the LBD modulate gating kinetics and subunit dimerization (Lomeli et al., 1994; Seeburg, 1996; Greger et al., 2006). Both impact on secretion of recombinant A2 from the endoplasmic reticulum (ER), where prolonging ER residence facilitates heteromeric assembly (Sukumaran et al., 2012; see also Coleman et al., 2010). Whether this mechanism contributes to the biogenesis of native AMPARs has not been addressed.

Here we show that alternative splicing in the LBD is subject to regulation. Chronic reduction of activity in hippocampal slice cultures results in changes at the flip/flop (i/o) cassette. Altered RNA splicing occurs for A1 and A2 in the CA1 subfield but not in CA3, implying cell-autonomous splicing regulation. Characterization of AMPARs after activity deprivation reveals changes in pharmacology and kinetics of extrasynaptic receptors, culminating in increased response fidelity. A functional switch is also evident at CA1 synapses, which cannot be explained by a direct effect of mRNA processing (Mosbacher et al., 1994) but rather by splice variant-driven receptor remodeling. These data reveal homeostatic regulation of short-term plasticity (STP) and suggest the existence of a dynamic mechanism readjusting AMPAR kinetics in the face of altered neuronal activity.

## Results

### AMPAR mRNA Reprogramming after Activity Deprivation

**Figure 1:**
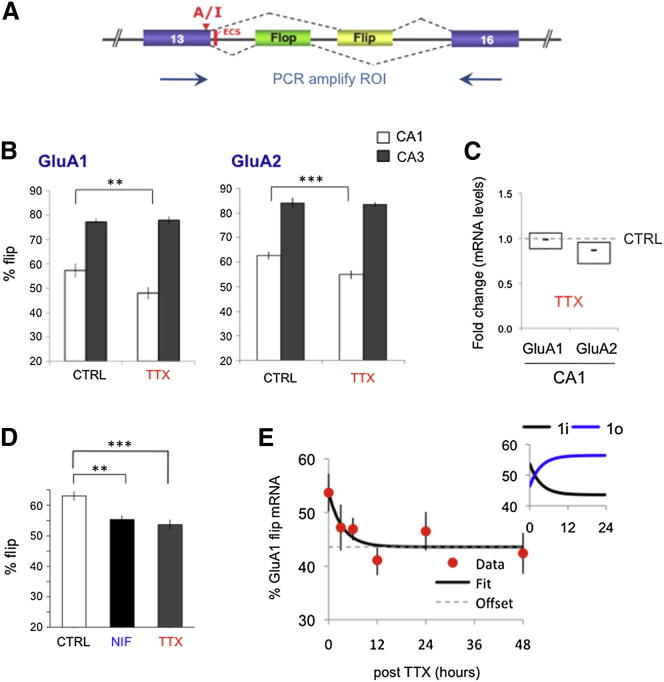
Activity-Dependent Changes in GluA1 and GluA2 RNA Processing Localized to Hippocampal CA1 (A) Schematic of *gria2* exons 13–16; the region of interest (ROI) amplified from cDNA encodes the i/o exons and the A/I (adenosine-to-inosine) RNA editing site. The editing complementary sequence (ECS) forms a conserved pre-mRNA secondary structure encompassing the splice site. (B) Quantification of peak heights in CA1 sequence chromatograms. Plots show the abundance of flip splice variants for A1 and A2 as a fraction of total subunit mRNA for ±TTX. Quantification of splice variants is determined from mean peak height ratios for the first five alternatively spliced nucleotides. Two-tailed t test, ^∗∗^p < 0.005; ^∗∗∗^p < 0.0001. (C) TaqMan real-time PCR measurements (A1 or A2/Gapdh) reveal no detectable differences between A1 and A2 mRNA expression after TTX relative to CTRL (dashed line). (D) Reduction of A2 flip levels in response to chronic nifedipine and to chronic TTX. ANOVA, p < 0.001; Dunnett’s multiple comparison (^∗∗^CTRL versus NIF; ^∗∗∗^CTRL versus TTX). (E) Time course of i/o splicing changes in response to TTX. Slices were harvested 0, 3, 6, 12, 24, and 48 hr post-TTX. The sample size was 9–17 slices/time point. The amount of A1i as a percentage of total A1i/o was determined from peak measurements of sequence traces. Data points were fit with a single exponential (y = A*e*^–τ/t^ + c). Since alternative splicing of i/o exons is mutually exclusive and overall A1 levels remain unchanged (C), a decrease in A1i is accompanied by an increase in A1o mRNA (inset).

AMPAR i/o splicing is segregated in rodent hippocampus—flip isoforms dominate in CA3, whereas CA1 neurons express predominantly flop (Sommer et al., 1990). This segregation is also apparent in RNA from rat organotypic slice cultures (see Figures S1A and S1B available online). This subfield-specific RNA profile will mostly reflect AMPAR expression in hippocampal pyramids since these cells make up approximately 90% of neurons in CA1 (Mishchenko et al., 2010; Olbrich and Braak, 1985; see Supplemental Information). Upon chronic activity deprivation (48 hr) with the Na^+^-channel blocker tetrodotoxin (TTX), levels of A1i and A2i transcripts diminish significantly in CA1, relative to untreated controls (Figure 1B). Since alternative splicing of i/o exons is mutually exclusive (Figure 1A) and overall A1 and A2 transcript levels are unaltered (Figure 1C), silencing with TTX leads to a concomitant upregulation of flop isoforms (Figure 1E, inset). Interestingly, RNA recoding at the i/o cassette is restricted to the CA1 subfield, i.e., is not apparent in CA3 (Figures 1B, S1B, and S1C) and is reversible—TTX washout reversed the processing pattern back to control (Figure S1F). Therefore, AMPAR alternative splicing is regulated in a reversible and subfield-specific manner, bearing hallmarks of homeostatic regulation.

Alternative splicing can be subject to control by external cues, in particular Ca^2+^ fluctuations (Xie, 2008). To test whether this is true for the i/o cassette, we blocked two major routes of external Ca^2+^ influx, NMDARs and L-type Ca^2+^ channels, the latter of which have been implicated in synapse-to-nucleus signaling (Thiagarajan et al., 2005; Wheeler et al., 2008). Whereas NMDAR block by chronic AP-5 treatment did not alter the balance of i/o splicing (data not shown), nifedipine (NIF) block of Ca^2+^ channels reduced levels of A2i, approaching values post-TTX (p < 0.05; ANOVA; Figure 1D), revealing regulation of the i/o cassette via Ca^2+^ through L-type channels.

### The Predominance of A1/A2 Heteromers Is Preserved after Chronic TTX

We next investigated the time course for alterations in RNA processing. The A2 mRNA half-life (t_1/2_) was ∼8–12 hr (data not shown), whereas alterations in i/o mRNA splicing were apparent ∼4 hr after TTX treatment and plateaued ∼24 hr post-TTX (A2i t_1/2_ ∼4.0 hr; Figures S1D and S1E). The A1 mRNA pool turned over more rapidly with i/o splicing changes already apparent ∼2 hr post-TTX (A1i t_1/2_ ∼2.4 hr; Figures 1E and S1E). This implies that 24 hr after TTX, recoded AMPAR mRNA predominates (see also Figure S7). To allow for sufficient protein turnover, we recorded AMPAR responses 48 hr post-TTX.

Hippocampal pyramids express mRNA for A1, A2, and A3 (Geiger et al., 1995; Tsuzuki et al., 2001), with A1/A2 heteromers predominating (Lu et al., 2009). To determine whether TTX treatment had an effect on subunit stoichiometry, we assessed AMPAR subunit composition. Low calcium permeability (*P*_Ca_/*P*_Na_; p > 0.05, two-tailed t test) and the near unity rectification indices (RIs, *g*_+10_ / *g*_−40_) of current-voltage (I/V) relationships were not different between the conditions (p > 0.05, Mann-Whitney U) (Figures S2A and S2B; Table 1). Therefore, A2-containing receptors prevail post-TTX. To determine whether A2 coassembled with A1 or A3, we used the polyamine toxin PhTx-74, which selectively blocks A1/A2 heteromers (Nilsen and England, 2007). Subunit selectivity could be confirmed in HEK293 cells expressing γ-8, a transmembrane AMPAR regulatory protein (TARP) (data not shown) (Rouach et al., 2005). When applied to CA1 patches from control slices, PhTx-74 almost completely attenuated currents and this inhibition was preserved after chronic TTX (p > 0.05, two-tailed t test; Table 1), indicating that A1/A2 heteromers remain the predominant AMPAR after activity blockade (Figures S2C and S2D).

### Alteration in Cyclothiazide Efficacy Suggests Changes in Splice Form Expression

A relative increase of flop mRNA is observed after TTX (Figures 1B and 1E, inset), which was unexpected as recombinant flop varieties are associated with more rapid desensitization kinetics (Jonas, 2000; Mosbacher et al., 1994). However, no significant changes in miniature excitatory postsynaptic current (mEPSC) decay kinetics were observed (p > 0.17, KS test; Figures S3C and S3F), in accord with previous studies (Kim and Tsien, 2008; Turrigiano et al., 1998). Similarly, entry into desensitization during prolonged glutamate application to excised patches was not significantly different (p > 0.05, two-tailed t test) (Table 1; Figure S4A, left).

**Figure 2:**
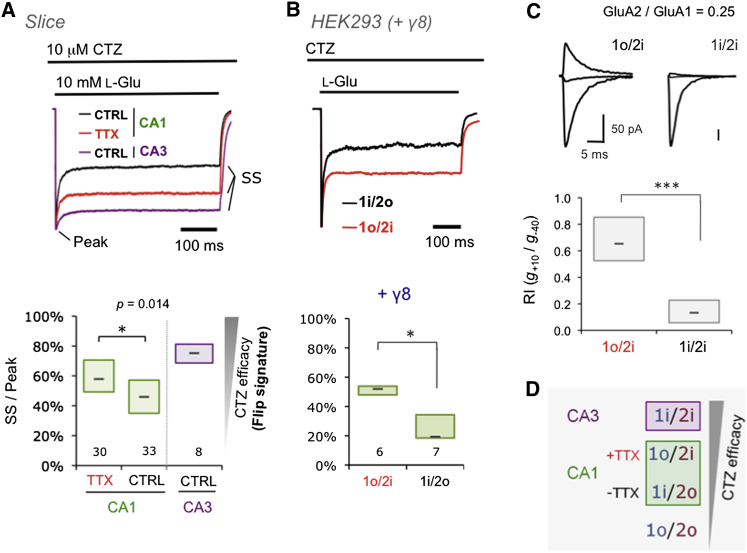
Remodeling of A1/A2 Splice Variant Combinations by Activity Deprivation (A) Current traces showing CTZ (10 μM) potentiation of the steady-state (SS) AMPAR current evoked with a 500 ms application of L-Glu (10 mM) (top). Traces are normalized to the peak current. The CTZ efficacy for patches from CA3 pyramidal cells is higher than that of CA1. CTZ efficacy in CA1 is elevated after 48 hr TTX. Data are summarized for all slices (bottom). The number below each box indicates the number of patches. Mann-Whitney U test, ^∗^p < 0.05. (B) The same experiment as described for (A) (but with 20 μM CTZ). Patches were pulled from HEK293 cells transfected with cDNAs to express A1i/A2o and A1o/A2i and γ-8. Heteromerization was confirmed by calculating rectification index (RI, see below in C; only patches with RI > 0.7 were used). Mann-Whitney U test, ^∗^p < 0.05. (C) Subunit variant A2i is more effectively recruited by A1o. Heteromerization of A1i or A1o with A2i (R/G edited) expressed in HEK293T cells. cDNAs were cotransfected at an A1/A2 ratio of 4:1; a ratio limiting for A2 coassembly. I/V relationships (in the presence of 100 μM intracellular spermine) were quantified by determining the slope conductance (g) at +10mV and −40mV from the observed reversal potential and expressing these as a ratio, *g*_+10_ / *g*_−40._ RIs are summarized as box plots, numbers of patches for 1o/2i were n = 10 and were n = 12 for 1i/2i. Mann-Whitney U test, ^∗∗∗^p < 0.001. Representative current traces of responses to 100 ms application of 3 mM L-Glu are shown above (current responses at −70mV, 0mV, and +50mV are shown). (D) A model of activity-dependent abundance of A1/A2 splice variant combinations in hippocampus based on the observed changes in CTZ efficacy and differential CTZ affinity of AMPAR flip variants.

Since native AMPARs are associated with auxiliary factors, which modulate gating (Guzman and Jonas, 2010; Jackson and Nicoll, 2011), differences in kinetics of splice isoforms may only become apparent in response to multiple stimuli (Arai and Lynch, 1996). We employed two approaches to compare AMPAR responses before and after activity blockade: multipulse protocols and drugs that differentiate between AMPAR splice isoforms. Cyclothiazide (CTZ) selectively blocks desensitization of flip receptors (Partin et al., 1994) and distinguishes splice isoform expression in hippocampal subfields (Arai and Lynch, 1996). Surprisingly, at odds with the decreased flip expression phenotype, CA1 patches from TTX-treated slices displayed significantly greater CTZ efficacy than controls (Figure 2A). As expected, responses from CA3, where flip forms predominate (Figure S1C), featured the greatest attenuation of desensitization (Figure 2A; Table 1).

CTZ displays a greater potency for A1/A2 heteromers containing A2i than A1i (Fleck et al., 1996; Miu et al., 2001; Partin et al., 1994). A greater proportion of A1/A2 heteromers harboring A2i may thus explain the elevated CTZ efficacy after TTX. To test this, we probed CTZ efficacy of A1/A2 splice heteromers expressed in HEK293 cells; recordings were done in the presence of the TARPs γ-2 (data not shown) or γ-8 (Figure 2B). In addition to mimicking native receptors more closely, this was also of interest since TARPs have the capacity to modulate CTZ action on AMPARs (Tomita et al., 2006). A1/A2 heteromers containing A2i did display a greater CTZ efficacy than heteromers harboring A1i (in the presence of both, γ-2, or γ-8) (Figure 2B and data not shown). Thus, the increased CTZ efficacy after chronic TTX could be explained by a greater proportion of A1/A2 heteromers containing A2i (Figure 2D).

### Selective AMPAR Assembly Driven by Flip/Flop Splicing

AMPAR assembly is also impacted by i/o splicing (Brorson et al., 2004; Coleman et al., 2010; Greger and Esteban, 2007; Penn and Greger, 2009), which implies that the i/o switch could modulate heteromeric assembly. We therefore measured I/V relationships of A1/A2 splice combinations in the presence of intracellular spermine with limiting transfection levels of A2. (Figure 2C). A2 incorporation alleviates inward rectification at positive holding potentials, resulting in an increase of the RI, a marker for heteromerization competence. The nonidentical splice heteromer A1o/A2i indeed produced a larger fraction of functional heteromers (RI ∼0.7) when compared to the identical splice pair A1i/A2i (RI ∼0.1) (Figure 2C). This indicates that the A1o isoform, which is elevated rapidly after chronic activity deprivation (Figures 1B and 1E), is more effective in recruiting A2i into heteromers, in harmony with the CTZ data. This preference was also seen in the presence of γ-2 (Figure S4B). Enhanced assembly of the opposite splice heteromer A1i/A2o was also observed relative to the splice homomers, albeit to a lesser extent (p < 0.01; ANOVA) (Figure S4B). These data reveal that A1o/A2i is the preferred subunit combination.

A1 protein transits through the secretory pathway more rapidly than A2. A2 accumulates in the ER and is thus saturating for heteromeric assembly at the subunit expression levels observed in our slices (Greger et al., 2002). The speedier A1 turnover rates in the ER together with the more rapid onset of splicing changes (to a flop:flip ratio of 1.4, relative to 0.9 seen under control conditions; Figure S7B) are expected to increase A1o levels in the early phases post-TTX. This relative and more rapid increase of A1o in TTX would have greater capacity to drive assembly of A1o/A2i heteromers (Figures 2C, 2D, and S7).

### Increased AMPAR Response Gain in Response to Reduced Activity

**Figure 3:**
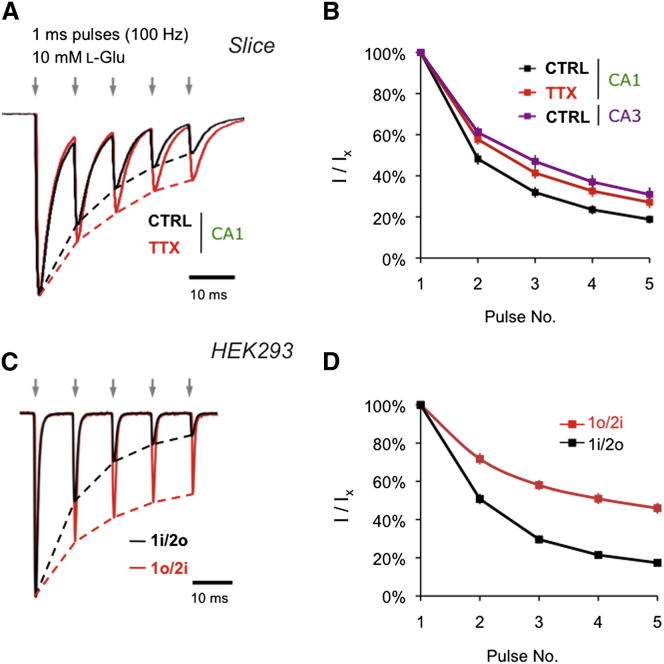
Distinct Kinetic Properties of Remodeled A1/A2 Receptors (A) Brief-pulse desensitization of AMPAR responses during a 100 Hz application (1 ms pulses) of L-Glu (10 mM). Traces are normalized to the peak current of the first pulse. Activity deprivation was associated with less depression of current amplitudes during the burst. (B) Summary of the group data for the assay in (A). Quantification of the burst responses by normalizing each response to the initial response (I_x_ / I_1_) is shown. Since responses summated, the current amplitude for each peak response was measured from the local preceding baseline (see Figure S4A). Number of patches for each treatment was 15 (CA1: CTRL), 15 (CA1: TTX), and 4 (CA3: CTRL). Values represent the mean ± SEM. (C) The stimulation protocol from (A) (train of 1 ms pulses of 10 mM L-Glu at 100 Hz) applied to recombinant A1/A2 heteromers. Note the faster AMPAR deactivation compared to CA1 patches caused by the absence of auxiliary subunits. (D) Group data for the assay in (C) as described in (B). Number of patches presented here is 6 for A1o/A2i and 6 for A1i/A2o.

**Figure 4:**
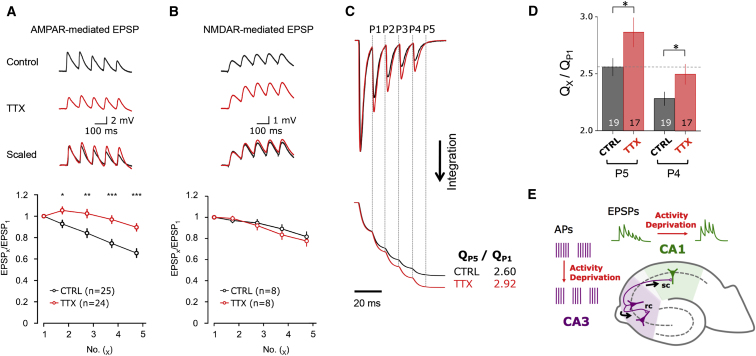
Reduced Depression of Synaptic AMPAR Responses and Increased Charge Transfer of Surface AMPARs during Burst Stimuli after Activity Deprivation (A) Repetitive Schaffer collateral stimulation (five pulses at 10 Hz, recorded at room temperature) caused depression of AMPAR-mediated EPSPs in control slices, which was absent in TTX. The values for EPSP1 were 5.1mV ± 0.4mV (n = 25; CTRL) and 4.1mV ± 0.4mV (TTX; n = 24; p > 0.05, two-tailed t test). Data are shown as mean ± SEM (CTRL: n = 25; TTX: n = 24); ^∗^p < 0.05, ^∗∗^p < 0.01, ^∗∗∗^p < 0.001, t test. (B) NMDAR-mediated EPSPs do not show changes in response to repetitive stimulation of afferent fibers post-TTX. Since responses summated, the voltage amplitude for each peak response was measured from the local preceding baseline. Data are shown as mean ± SEM (CTRL: n = 8; TTX: n = 8). (C) Scaled current responses to 100 Hz trains of L-Glu (10 mM) to CA1 patches (top) and their integration to illustrate increased charge transfer after chronic TTX (bottom). The relative changes in charge transfer for the whole train were quantified by measuring the charge (Q) after the last pulse (P5) normalized to the charge after first pulse (P1). The ratios for the example traces are 2.60 for CTRL and 2.92 for TTX. (D) Summary data for normalized charge transfer (Q_X_ / Q_P1_) using the analysis shown in (C). The charge transfer was significantly higher after chronic TTX (p < 0.05, two-tailed t test). Number of patches is indicated at the base of the column. (E) Model for the role of globally remodelled AMPARs with reduced brief-pulse desensitization in maintaining charge transfer for shorter input bursts, which result from activity deprivation (Kim and Tsien, 2008). APs, action potentials; EPSPs, excitatory postsynaptic potentials; sc, Schaffer collaterals; rc, recurrent collaterals.

Kinetic differences between alternative splice forms of native AMPARs can be revealed by applying multiple pulses of agonist (Arai and Lynch, 1996). We applied trains of glutamate (five 1 ms pulses; 100 Hz) to CA1 and CA3 patches, which mimic spike firing patterns of Schaffer collateral inputs during CA3 pyramidal cell bursting (Spruston and McBain, 2007). AMPARs in CA3 feature less brief-pulse desensitization and reduced depression due to the prevalence of flip receptors, which desensitize slower and recover from desensitization more rapidly (Arai and Lynch, 1996; Mosbacher et al., 1994). Similarly, in our cultures, response fidelity was more pronounced in CA3 than in CA1 (Figure S4A, right). Fidelity of somatic AMPARs in CA1 was elevated after TTX treatment at both 100 Hz (Figures 3A and 3B) and at 50 Hz (data not shown). Since in A1/A2 heteromers A2i (but not A1i) dominate desensitization kinetics (Mosbacher et al., 1994) (Figure S4C), these results further imply a greater contribution of A2i-harboring heteromers after chronic TTX. Moreover, the response pattern to 100 Hz trains after TTX also resulted in an almost 15% increase in charge transfer (p < 0.05, two-tailed t test; Figures 4C and 4D); this increased gain could compensate for the dampened network activity post-TTX (Kim and Tsien, 2008).

To test the potential reshuffling of A1/A2 splice forms after TTX more directly, we subjected A1/A2 variants expressed in HEK293 cells to the same protocol. A1o/A2i heteromers displayed greater response fidelity than A1i/A2o receptors (Figures 3C and 3D), mimicking the behavior of native AMPARs post-TTX (Figures 3A and 3B). AMPAR desensitization is also affected by R/G editing (Lomeli et al., 1994). However, nonedited (A2o-R) and edited (A2o-G) looked identical in this assay (Figure S4C). Moreover, response properties of the pure flip combination (A1i/A2i) closely matched the A1o/A2i heteromer, arguing against a contribution from the A1i splice form (Figure S4C). In the presence of TARPs γ-2 or γ-8, gating kinetics are slowed, the relative difference between the splice heteromers was however preserved and increased response fidelity of A1o/A2i receptors was still evident (Figure S4D). In sum, selective incorporation of A2i into A1/A2 heteromers after TTX results in AMPARs with enhanced responsiveness to burst-like stimulations.

### Involvement of AMPAR Auxiliary Factors Is Not Altered after Chronic TTX

Since TARPs modulate receptor kinetics, we directly assayed potential changes in expression of these cofactors in response to TTX (Figure S5A). This analysis did not uncover differences in TARP expression between control and TTX for γ-2, γ-3, and γ-8 (Figures S5B and S5C).

TARPs dose-dependently slow deactivation kinetics and increase the slow component of AMPAR desensitization (Jackson and Nicoll, 2011; Tomita et al., 2005). We could not discern differences in deactivation time constants (p > 0.05, two-tailed t test; Table 1) or the amplitude of the slow component of desensitization (p > 0.05, two-tailed t test), arguing against a significant increase in TARP contribution after TTX treatment. Similarly, kainate efficacy was comparable between TTX-treated and control slices (p > 0.05, two-tailed t test; Table 1). Lastly, efficacy of the noncompetitive AMPAR antagonist GYKI-52466, which is increased by TARPs (Cokić and Stein, 2008), was very similar between the two conditions (p > 0.05, two-tailed t test; Table 1; Figure S5D).

Another group of AMPAR cofactors, referred to as cornichons (CNIH2 and CNIH3), also slow down the kinetics of channel gating (Schwenk et al., 2009). Analysis of their expression levels (Figures S5E–S5G) did not show differences between control and TTX conditions. Together, an altered contribution of AMPAR cofactors post-TTX is not apparent.

### Reduced Depression of AMPAR EPSPs Follows TTX Silencing

Silencing with TTX gives rise to compensatory adjustments at synapses (Turrigiano, 2008), including an upregulation of AMPAR mEPSC amplitudes in CA1 (Kim and Tsien, 2008), which we also observe (Figures S3A–S3D and S3F). To investigate whether reduced depression of AMPAR responses to burst-type stimulations (Figures 3A and 3B) is expressed at synapses, we recorded CA1 excitatory postsynaptic potentials (EPSPs) evoked by stimulating Schaffer collaterals (five pulses at 10 Hz). Whereas CA1 neurons from control slices exhibited a marked depression, responses faithfully followed the train post-TTX: (EPSP2/1: CTRL: 0.93 ± 0.04, n = 25; TTX: 1.05 ± 0.05, n = 24, p < 0.05; EPSP5/1: CTRL: 0.65 ± 0.04, n = 25; TTX: 0.90 ± 0.04, n = 24, p < 0.01; Figure 4A). A similar pattern was obtained by increasing the frequency to 50 Hz at elevated recording temperature (34°C–37°C) (Figure S6A).

The burst-type stimulations used are an extension of paired-pulse protocols, which are used to evaluate presynaptic changes such as release probability (Pr) (Pozo and Goda, 2010; Zucker and Regehr, 2002). Limiting transmitter release by lowering the Ca:Mg ratio caused facilitation in control slices (Figure S6Cii). We explored whether presynaptic effects contributed to the altered EPSPs post-TTX. First, we recorded NMDAR-mediated EPSP bursts. No differences between control and TTX were evident for the NMDAR component at 10 Hz (EPSP2/1: CTRL: 0.97 ± 0.03, n = 8; TTX: 0.99 ± 0.03, n = 8, p = 0.6; EPSP5/1: CTRL: 0.82 ± 0.05, n = 8; TTX: 0.78 ± 0.05, n = 8 p = 0.58) (Figure 4B). As a more direct measure for changes in Pr, we determined the rate of use-dependent block of NMDAR responses by MK-801, which is proportional to Pr (Hessler et al., 1993). However, MK-801 block was not significantly different between control and TTX (p > 0.1, two-tailed t test; Figure S6B). If anything, we observed a trend toward faster block after TTX—implying a greater Pr or higher glutamate concentration in the synaptic cleft, which would be associated with greater depression rather than the reduced depression in TTX (Figure S6Cii) (Zucker and Regehr, 2002). This was confirmed by using the low-affinity, competitive AMPAR antagonist γ-DGG, which suppresses AMPAR responses more effectively under reduced glutamate concentrations (Lei and McBain, 2004; Shen et al., 2002; Wadiche and Jahr, 2001). Again, this assay showed no significant difference between the two conditions, but pointed to a trend-wise increase in synaptic glutamate after TTX (as γ-DGG was less effective in suppressing AMPAR responses) (Figure S6Ci). Therefore, the reduced depression of the AMPAR response after chronic TTX observed at somatic and synaptic sites (Figures 3A and 4A) is consistent with a global, RNA-based AMPAR remodeling mechanism.

## Discussion

Here we present a mechanism for synaptic homeostasis—the expression of kinetically different AMPARs after activity deprivation, which increases transmission fidelity in response to repetitive stimulation. Subunit remodeling is triggered by an alteration of splice variant mRNA, which is regulated by activity in a reversible, subfield-specific manner. As a result, an elevated contribution of A1o/A2i heteromers is apparent (Figure S7), which compensates for the loss of synaptic drive in TTX. Positions recoded by i/o splicing line the LBD dimer interface, where they have been implicated in modulating assembly of recombinant AMPARs (Brorson et al., 2004; Greger and Esteban, 2007; Penn and Greger, 2009). Such a mechanism is expected to be metastable (a function of mRNA turnover rates) and to act globally and could thus affect other forms of synaptic plasticity.

### Regulated RNA Processing in CA1

TTX treatment reduces CA1 flip levels, which remain the predominant isoform in CA3. Factors regulating different RNA processing in CA1 and CA3 have not been elucidated. The general splicing factors SF2 and SC35, which favor the expression of flop variants (Crovato and Egebjerg, 2005), were no different in their mRNA levels between CA1 and CA3 (data not shown). A selective involvement of SRp38 in facilitating expression of the flip exon has been highlighted (Feng et al., 2008; Komatsu et al., 1999), where reduced levels of SRp38 result in flop inclusion (Feng et al., 2008). However, analysis of SRp38 mRNA levels did not reveal differences between CA1 and CA3 (in mouse and rat; I.H.G. and A.B., unpublished data). SRp38 protein is activated by phosphorylation but acts as a splicing repressor upon dephosphorylation (Feng et al., 2008), which has only been noted under specific circumstances such as heat shock (Shin and Manley, 2002). SRp38 phosphorylation levels in CA1 and CA3 were unaltered (I.H.G. and A.B., unpublished data). Therefore, candidate splicing factors remain elusive.

### AMPAR Assembly Driven by Altered RNA Processing in the LBD

A summary of the events leading to activity-mediated assembly is outlined in Figure S7A; both mRNA and protein turnover will contribute: A1i mRNA turns over more rapidly, thus A1o transcripts will be enriched relative to A2o in the earlier phases after TTX treatment. In addition, A1 protein has a shorter ER half-life in neurons, whereas A2 stably resides in the ER (Greger et al., 2002). Therefore, in response to TTX, A1o protein will emerge earlier and will sample from a mixed pool of A2 splice forms, preferentially recruiting A2i into heteromers. Here we show that this altered expression of splice variants affects preferential assembly of native AMPARs. Whether the i/o assembly drive is mediated directly by selective LBD association affinities or is predominantly linked to functional properties (Penn et al., 2008) requires further investigation. In support of the latter, the higher ER residency of A1o (Coleman et al., 2010) (which increases after TTX) would boost heteromeric assembly of the favored A1o/A2i combination. Regarding the former, analytical ultracentrifugation of isolated LBDs from A2i and A2o do not suggest tighter dimerization between splice heteromers (I.H.G., unpublished data). Whether differences could be revealed with splice heteromers of A1/A2 LBD dimers or a role of the R/G editing site, which also changes in response to TTX, remains an open question.

### Homeostasis via AMPAR Short-Term Plasticity

Homeostatic control operates via diverse, parallel mechanisms, both intrinsic and synaptic (Turrigiano, 2008). To date, postsynaptic homeostatic plasticity almost exclusively involves changes in the number of AMPARs. The finding that the balance of i/o splice isoforms has the capacity to modulate expression of functionally distinct AMPAR heteromers provides additional plasticity to synaptic homeostasis. The expression of AMPARs with altered kinetics will increase postsynaptic efficacy under conditions of network silence, while we have shown that the involvement of a prominent presynaptic component seems less likely. Since TTX treatment reduces burst duration in CA3 (Kim and Tsien, 2008), AMPAR remodeling in CA1 will facilitate faithful information processing. Whether physiologically relevant activity such as brain oscillations can trigger splicing-mediated subunit remodeling and to what extent this splicing regulation affects AMPAR signaling in other circuitries remains to be elucidated.

## Experimental Procedures

### Slice Cultures

All procedures were carried out in accordance with UK Home Office regulations. Transverse hippocampal slices (300–400 μm thick) were cut from postnatal day 5 Sprague-Dawley pups and cultured for at least 3 weeks prior to drug treatments.

### Molecular Biology

RNA was isolated from hippocampal subfields with Trizol (Invitrogen), DNaseI treated, and random primed with reverse transcriptase; resulting cDNA served as template for PCR amplifications of the regions of interest (ROIs). Products were Sanger sequenced, and peak heights in chromatograms were measured to determine splice variant ratios.

### Electrophysiology

Outside-out patches were excised from pyramidal cells and AMPAR conductances were activated via ultra-fast L-Glu application. Synaptic AMPAR EPSPs were evoked by Schaffer collateral fiber stimulation.

Refer to Supplemental Experimental Procedures for details.

## Figures and Tables

**Table 1:** Comparing Functional and Pharmacological Properties of AMPARs in CA1 ± TTX

		CTRL				TTX			
		Median	Mean	SEM	n∗	Median	Mean	SEM	n∗
Deactivation	τ (ms)	2.8	2.8	0.1	19 (12)	2.7	2.8	0.1	16 (11)
Desensitization	τ_fast_ (ms)	7.6	7.8	0.4	37 (22)	7.4	7.8	0.4	37 (19)
	Amp_fast_	62.6%	61.8%	2.1%	37 (22)	58.8%	57.1%	1.8%	37 (19)
	τ_slow_ (ms)	28.6	29.9	1.6	37 (22)	26.0	28.9	1.5	37 (19)
	Amp_slow_	37.4%	38.2%	2.1%	37 (22)	41.2%	42.9%	1.8%	37 (19)
	τ_weighted_ (ms)	14.2	15.9	0.8	37 (22)	15.5	16.6	0.8	37 (19)
	Percentage of desensitization	98.0%	97.6%	0.3%	37 (22)	98.1%	97.3%	0.4%	37 (19)
Rectification Index (*g*_+10_ / *g*_−40_)∗∗		0.94	0.94	0.02	14 (8)	0.95	1.00	0.02	13 (7)
*P*_Ca_ / *P*_Na_∗∗		0.100	0.102	0.003	5 (5)	0.099	0.099	0.003	5 (5)
Percentage Block of I_Glu,Peak_ by 100 μM Philanthotoxin 74∗∗		95.8%	95.0%	1.3%	6 (5)	92.9%	93.9%	1.5%	5 (4)
SS/Peak in 10 μM cyclothiazide		45.9%	46.8%	3.0%	33 (23)	57.9%	58.0%	3.1%	30 (19)
I_KA_/I_Glu,Peak_∗∗∗		0.14	0.13	0.02	4 (2)	0.11	0.11	0.01	5 (2)
Percentage of Inhibition of I_Glu,Peak_ by 10 μM GYKI 52466∗∗∗		46.8%	45.8%	1.4%	9 (6)	48.9%	48.4%	1.8%	5 (5)

∗ The sample size is the number of cells. The number of slices is indicated in brackets.

∗∗ Experiments were carried out with 1 mM QX-314 Cl and 100 μM spermine added to the intracellular solution.

∗∗∗ Concentrations used for GYKI-52466 (10 μM) and kainate (KA; 500 μM) are nonsaturating for hippocampal neurons (Paternain et al., 1995; Jonas and Sakmann, 1992). Related to Table S1.

## Supplemental Information

Document S1. Figures S1–S7, Table S1, and Supplemental Experimental Procedures
