# Supplementary material for: Activity-Mediated AMPA Receptor Remodeling, Driven by Alternative Splicing in the Ligand-Binding Domain
Source: Neuron. 2012 Nov 8;76(3):503–10. doi: 10.1016/j.neuron.2012.08.010 (PMC3500689; doi:10.1016/j.neuron.2012.08.010)
Supplement: Document S1. Figures S1–S7, Table S1, and Supplemental Experimental Procedures [file mmc1.pdf]

Neuron, Volume *76*

Supplemental Information

Activity-Mediated AMPA Receptor Remodeling, Driven by Alternative Splicing in the Ligand-Binding Domain

Andrew C. Penn, Ales Balik, Christian Wozny, Ondrej Cais, and Ingo H. Greger

Supplementary Figures and Legends

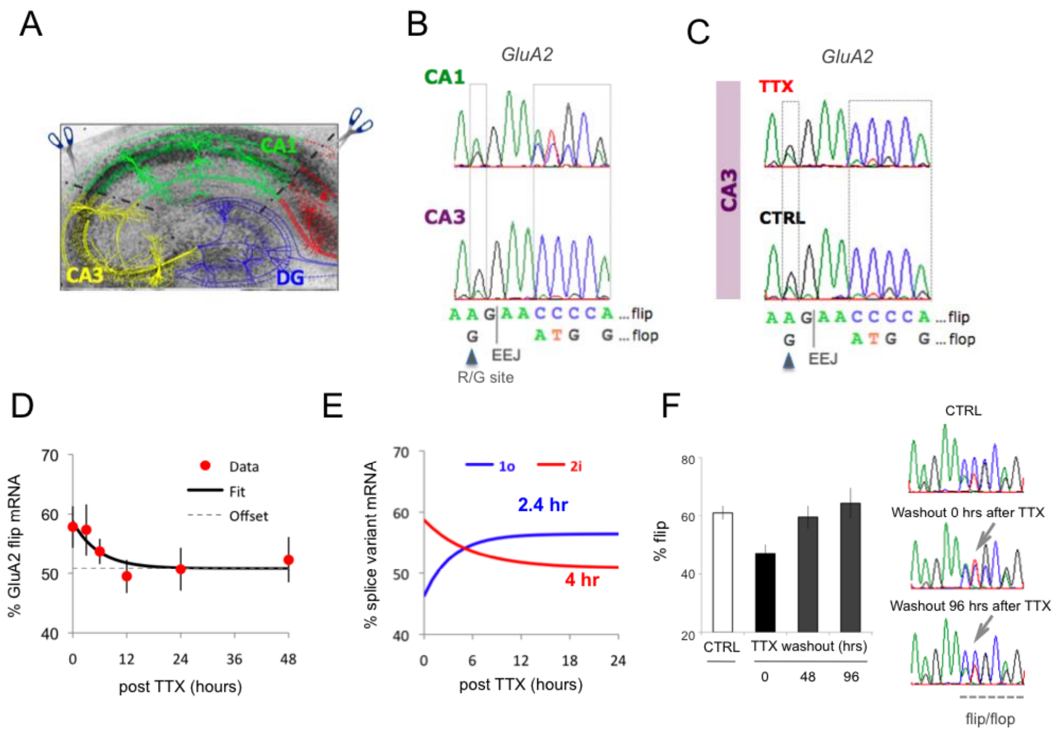

Figure S1

Figure S1 (related to Figure 1). Differential RNA processing in hippocampus CA1 and CA3 in response to TTX

A. Nissl-stained hippocampal slice (3 months in culture). The major subfields are superimposed onto the slice and are color-coded (Subiculum [S], red; CA1, green; CA3, yellow and dentate gyrus [DG], blue) (see Supplemental Text).

B. Sequence traces of PCR-amplified GluA2 cDNA from CA1 and CA3 of control slices, the mixed peaks correspond to R/G editing and i/o splicing

(boxed). A clear difference between these subfields with regard to editing (Balik et al., 2012) and i/o splicing is apparent. CA3 is predominantly edited to G and mostly expresses flip.

C. Sequence traces of PCR-amplified GluA2 cDNA from CA3 control and TTX-treated slices. Drug treatment was for 48 hours.

D. Time course of changes of flip/flop splicing induced by chronic TTX treatment. Slices were harvested at various time points (0, 3, 6, 12, 24 and 48 hrs) after TTX application. The sample size was 8-17 slices for each time point. The amount of GluA2i as a percentage of total GluA2i/o was determined from peak measurements of sequence traces. Data points were fit with a single exponential ($y = Ae^{-\tau/t} + c$) in Axograph 4.8. A half-life ($t_{1/2}$) of 4.0 hours was determined from the time constant (τ) using the equation: $t_{1/2} = \tau \,.\, (-\ln(0.5))$.

E. Summary of GluA1o (blue curve) and GluA2i (red curve) mRNA turnover rates in response to TTX treatment.

F. Right: Representative sequence traces of PCR-amplified GluA2 cDNA demonstrate reversibility of RNA processing for 0 and 96 hrs of washout following a 48 hr TTX treatment. Recovery to control levels at the 96 hr time point is shown. Bar graph (left) summarizing quantification of GluA2 i/o splicing between CTRL and TTX washout samples determined from peak height ratios (n=3-4).

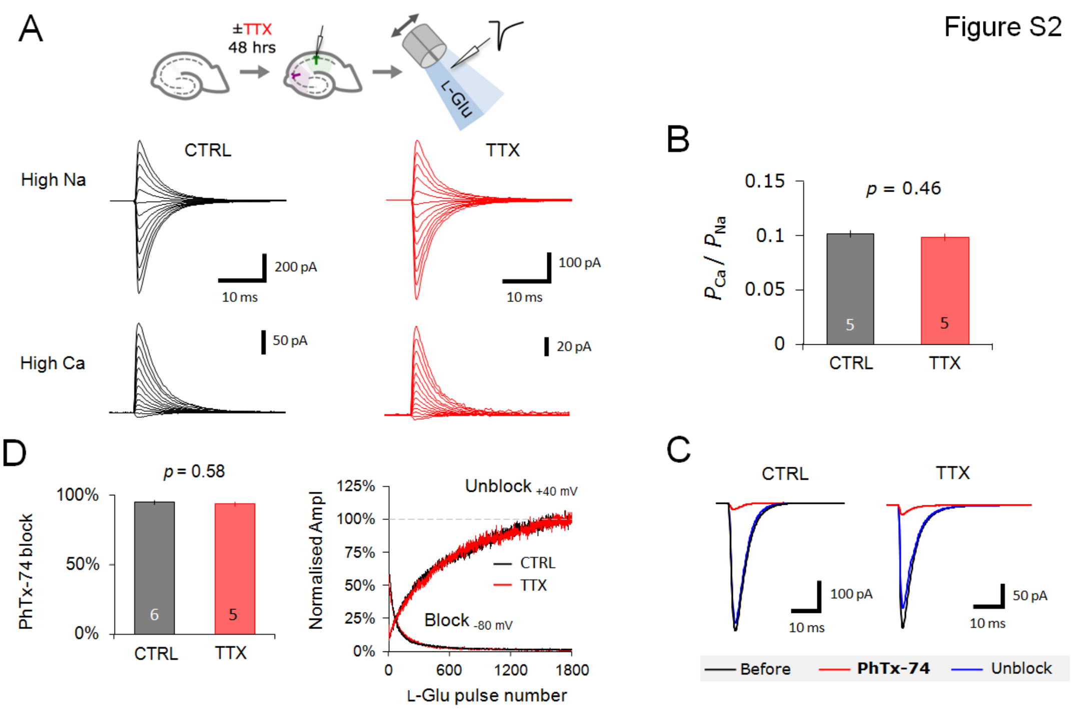

Figure S2 (related to Figure 2). Subunit composition of surface-expressed AMPARs in CA1 remain as A1/2 heteromers after activity deprivation

A. Outside-out patches were excised from CA1 pyramidal cells from slices following 48 hr CTRL (black) and chronic TTX (red) treatments and AMPAR currents were activated with 1 ms applications of L-Glu (3 mM) in high sodium (Na, top) and high calcium (Ca, bottom) extracellular solutions at holding potentials between -70 and + 50 mV (in 10 mV steps). NMDA receptors were blocked by supplementing extracellular solutions with 50 μM DL-AP5. The intracellular solution contained 100 μM added spermine to reveal rectification characteristic of A2-lacking receptors (see Supplemental Experimental procedures and Supplemental Text). Note the linear current-voltage relationship in high Na solution (from which the RI is calculated) and the very low reversal potential in high Ca solution indicative of A2-containing AMPAR heteromers.

B. There is no difference between the calcium permeability (P_{Ca}/P_{Na}) of AMPARs between the two treatments indicating that most contain the A2 subunit. The number at the base of each column indicates the number of patches. Values represent the mean ± SEM. The *p*-value is derived from a two tailed t-test.

C. Outside-out patch recordings of peak AMPAR currents activated with 1 ms applications of L-Glu (3 mM) before and after block by 100 μM Philanthotoxin (PhTx) 74 (left, see Methods). When co-expressed with stargazin (γ-2, Nilsen and England, 2007) and γ-8 (unpublished data, O.C.), recombinant A1/2 (but not A2/3) heteromers are blocked by 100 μM PhTx 74. In CA1 patches, ~ 95% block could be achieved for both CTRL (left) and TTX (right)-treated slices (48 hours) indicating that the A2-containing surface receptors were heteromers with A1 and that this did not change significantly with treatment. More than 50 % of the original current amplitude could be recovered in all patches following washout, with the mean for each treatment being ~ 70%.

D. Summary graph of PhTx-74 results for TTX and CTRL (left). The number at the base of each column indicates the number of patches. The *p*-value is derived from a two-tailed t-test. The average time course of block (at -80 mV) and unblock (at +40 mV) normalized to the first pulse or the mean of the final 20 pulses, respectively (right). PhTx-74 block plateaued at the same level and relief of the block occurred at equal rate implying equal PhTx-74 affinity between CTRL and TTX. Together, the data indicate that surface expressed AMPARs in CA1 are A1/2 heteromers and remain so after chronic TTX treatment.

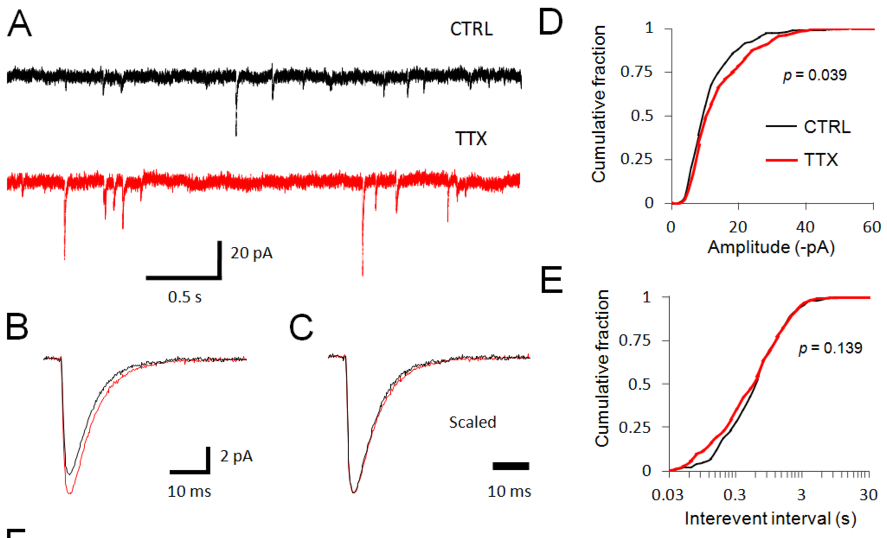

F

Summary of mEPSC data

Property	CTRL		TTX	
Amplitude (-pA)	10.41	± 0.78	11.93	± 0.93
Frequency (Hz)	1.61	± 0.29	2.08	± 0.56
20 - 80 % rise time (ms)	1.15	± 0.11	1.15	± 0.15
Decay time constant (ms)	5.62	± 0.62	6.09	± 0.51
Series resistance (MΩ)	14.4	± 2.0	12.8	± 1.4
Number of cells	6		6	

Figure S3

Figure S3 (related to Figure 4). Miniature EPSC recordings reveal that our slice cultures can undergo classical homeostatic plasticity

A. Representative recordings of CA1 pyramidal cell miniature EPSCs (mEPSCs) from organotypic slices at a holding potential of -70 mV following incubation ± 48 hr TTX.

B. Superposition of the mean mEPSCs for the two conditions indicate an increase in mEPSC amplitude after activity deprivation. Data represents 50 mEPSCs per cell: 6 cells for CTRL, 6 cells for TTX.

C. Peak-scaling of mean mEPSCs shown in B indicate the absence of any substantial change in the mEPSC rise or decay kinetics (bottom).

D. Cumulative frequency histogram plots for mEPSC peak amplitude. The distributions for CTRL and TTX conditions were compared with a two sample Kolmogorov-Smirnov test (two-tailed). mEPSC data represent 50 mEPSCs per cell: 6 cells for CTRL, 6 cells for TTX.

E. Cumulative frequency histogram plots for interevent interval (right). Since the distributions were better described by a lognormal model, the histogram

binning was calculated on the log transformed interevent intervals. The bin times were then back-transformed and the axis was log scaled. The interevent intervals for CTRL and TTX conditions were compared with the (distribution-free) two sample Kolmogorov-Smirnov test (two-tailed). A small increase in mEPSC frequency is apparent but does not reach statistical significance (see Figures S6B, C). mEPSC data represent 50 mEPSCs per cell: 6 cells for CTRL, 6 cells for TTX.

F. Table summarizing mEPSC and recording data. mEPSC data represent 50 mEPSCs per cell: 6 cells for CTRL, 6 cells for TTX. mEPSCs were detected with a 4 pA amplitude threshold. The results here were also reproduced in a separate experiment (unpublished data, A.C.P).

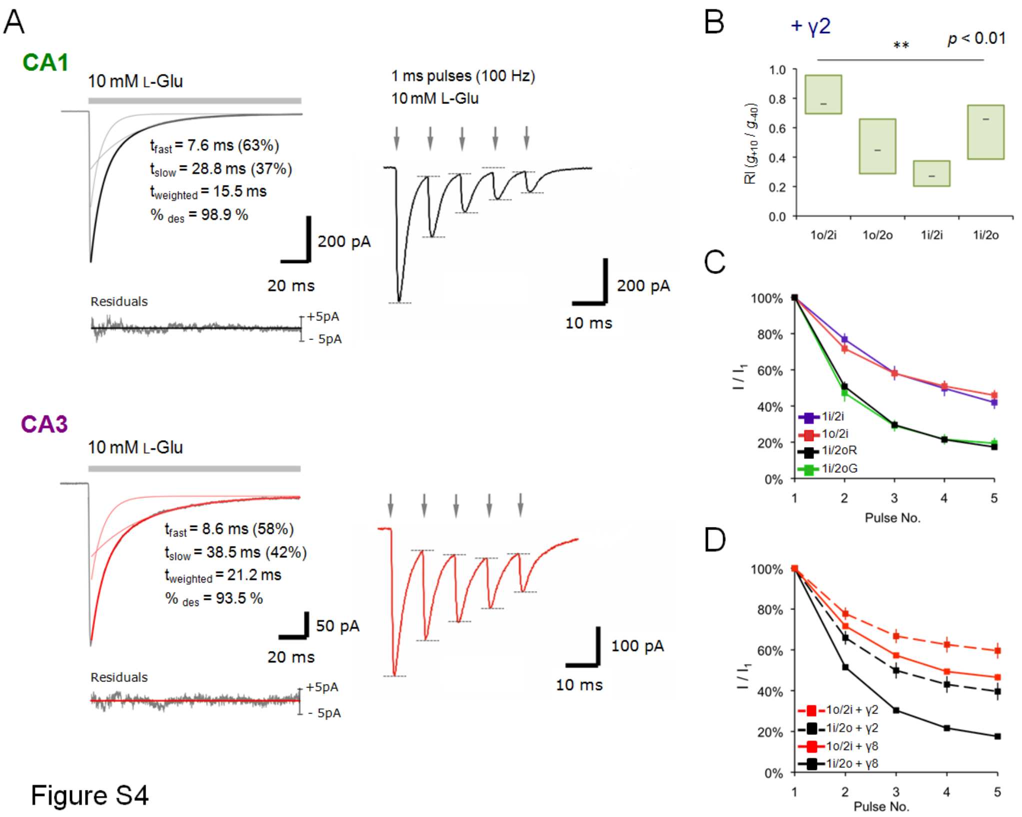

Figure S4

Figure S4 (related to Figure 3). Kinetics of native and recombinant AMPAR variants in response to burst stimuli

A. Representative current traces demonstrating faster entry into desensitization (left) and lower fidelity (right) of AMPAR responses to L-Glu application in patches from CA1 (top) than from CA3 (bottom) pyramidal neurons. The two component exponential fits and the residuals are shown. Response fidelity was assessed using a train of five pulses (1 ms) of L-Glu (10 mM) delivered at a frequency of 100 Hz. The current amplitude for each peak response was measured from the local preceding baseline (right top and bottom). Peaks and baseline points are marked with dashed lines.

B. Splice variant differences provide more effective assembly of GluA1/2 heteromers in γ-2 expressing HEK293 cells. This experiment was performed at a transfection ratio where GluA2 is limiting for the assembly of heteromers. Therefore, rectification index (RI) can be used as an indicator of assembly competence (see also Figure 2C). GluA1o preferentially recruits GluA2i. Furthermore, 1o/2i assemble more effectively than 1i/2o heteromers. The *p*-value was derived from the test-statistic of a one-way ANOVA.

C. GluA2 i/o splicing (and not GluA1 i/o splicing nor GluA2 R/G editing) is the main determinant of the response fidelity in this assay (see Supplemental Text). Summary graph for the responses of recombinant GluA1/GluA2 receptors to trains of stimuli (see Fig 4b,c). Number of patches is 6, 6, 6 and 5 for 1i/2i, 1o/2i, 1i/2oR and 1i/2oG, respectively.

D. The response fidelity of the 1o/2i splice variant combination to 100 Hz glutamate trains exceeds that of the 1i/2o in HEK293 cells expressing either γ-2 or γ-8. Number of patches is 6, 8, 9 and 10 for 1o/2i + γ-2, 1i/2o + γ-2, 1o/2i + γ-8 and 1i/2o + γ-8, respectively. The stimulation protocols and analysis were as shown in Figure 3. As in C and Figures 3C-D, only patches with RI > 0.7 were included.

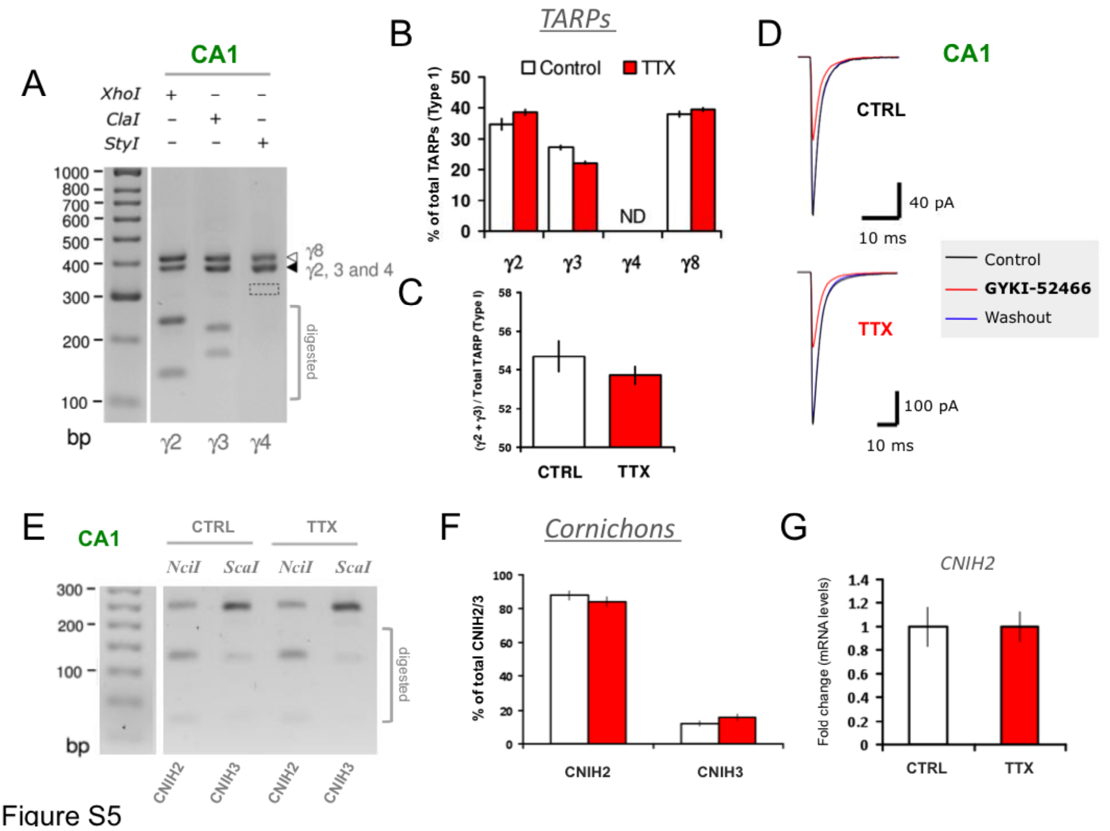

Figure S5

Figure S5 (related to Figure 2). Expression levels of AMPAR auxiliary factors remain constant after chronic TTX

A. CA1 expresses TARP γ-2, γ-3 and γ-8 mRNA. RT-PCR fragments amplified with panTARP primers were digested with the following restriction enzymes: γ-2 (*XhoI*), γ-3 (*ClaI*), γ-8 (*StyI*). Digestion products were loaded onto a 3% agarose gel and post-stained with ethidium bromide. Uncut γ-8 runs at a higher molecular weight than the other type-1 TARPs. The hatched box indicates the position of the γ-4 specific *StyI* digestion fragment from a rat brain control digest (not shown). γ-4 is below detection in these CA1 preparations.

B. Summary of the TARP expression data. The expression of type-1 TARPs is not markedly changed in response to TTX (red bars), compared to control (empty bars). Small (~ 5%) complementary changes in the expression of functionally redundant γ-2 and γ-3 are apparent. Error bars represent SEM. ND = not detected.

C. The relative expression of γ-2/3 vs. γ-8 in control and TTX treatments is similar. Error bars represent SEM. ND = not detected.

D. Activity deprivation does not modulate AMPAR antagonism by GYKI-52466, which could otherwise indicate changes in TARP association. Outside-out patch recordings of peak AMPAR currents activated with 1 ms applications of L-Glu (10 mM) before, during application and after wash-out of 10 μM GYKI-52466: a concentration equal to the IC_{50} for this drug on native hippocampal AMPARs (Paternain, Morales and Lerma, 1995). 50% block could be achieved for both CTRL (left) and TTX (right)-treated slices (48 hours) indicating equivalent efficacy of GYKI-52466 antagonism. Group data area summarized in Table 1.

E. Cornichons (CNIH2 and 3) mRNA levels remain constant. RT-PCR fragments amplified with panCNIH primers were digested with the following restriction enzymes: CNIH2 (*NciI*), CNIH3 (Sc*aI*). Digestion products were loaded onto a 2.5% agarose gel and post-stained with ethidium bromide. Uncut top bands represent relative proportions of CNIH3 and 2 in CTRL and TTX, respectively.

F. Summary of the CNIH2/3 expression data from panel E. The expression of CNIH2/3 is not markedly changed in response to TTX (red bars), compared to control (empty bars). Error bars represent SEM.

G. Sybr-green based quantitative PCR measurements reveal no detectable differences for CNIH2 mRNA expression between CTRL and TTX treated slices. n = 21 (CTRL); n = 17 (TTX).

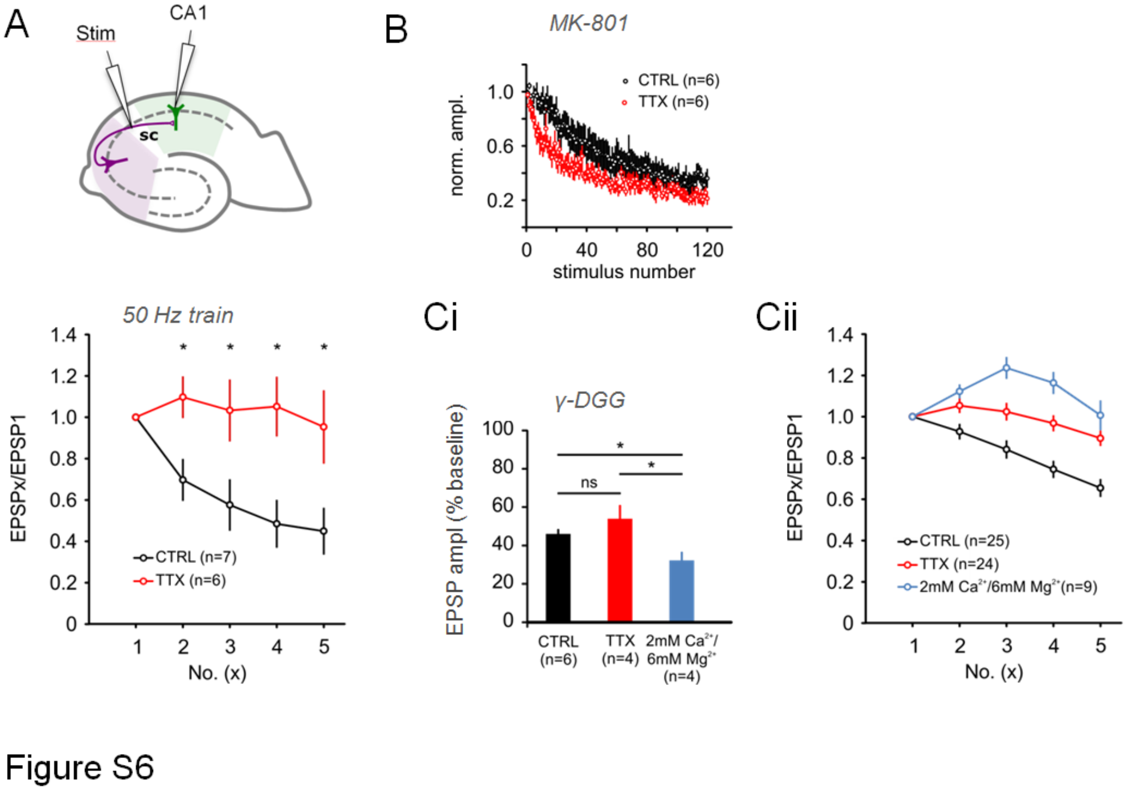

Figure S6 (related to Figure 4). Pre-synaptic changes do not explain the enhanced fidelity to burst stimuli post chronic TTX

A. At near physiological temperatures repetitive stimulation of Schaffer collaterals (5 pulses at 50 Hz) caused a pronounced depression of AMPAR-mediated excitatory postsynaptic potentials (EPSPs) in control slices (CTRL). In TTX-treated slices this depression was largely absent and EPSP amplitudes remain stable throughout the train similar to the responses following the 10 Hz stimulation recorded at room temperature (Figure 4). Data are shown as mean ± SEM; * $p < 0.05$, Student's t-test.
B. To test directly whether presynaptic release probability is altered after chronic deprivation of neuronal activity we measured the release probability using the NMDA channel blocker MK-801, which irreversibly blocks NMDARs after each synaptic release (Hessler et al., 1993; Rosenmund et al., 1993). During synaptic stimulation in the presence of MK-801 (40 μM), NMDAR-mediated synaptic responses decline reflecting the average release probability of these synapses. We found that the decay rate of postsynaptic responses as a function of stimulus number is not significantly altered following TTX treatment.

Results were fitted to a single exponential as a measure of the average release probability (CTRL: τ =110 ± 20 ms, ranging from 43 – 182 ms, n = 6; TTX τ=68 ± 11 ms, range 49 – 116 ms, n = 6; $p > 0.1$, Student's t-test)

C.

i. The low affinity competitive AMPAR antagonist γ-DGG was used to investigate the synaptic glutamate transient (Lei and McBain, 2004; Shen et al., 2002; Wadiche and Jahr, 2001). To provide evidence that the synaptic glutamate transient (as a measure of release probability) is unaltered following TTX-treatment we measured the degree of EPSP reduction after washing in γ-DGG (0.5 mM). Indeed, the EPSP amplitude is reduced to 0.46 ± 0.02 of its baseline values in control slices (CTRL: n = 6) and, similarly, to 0.54 ± 0.07 in TTX-treated slices (TTX: n = 4, $p > 0.3$, Student's t-test). Reducing the release probability and the synaptic glutamate transient by decreasing the extracellular Ca^{2+} concentration from 4 mM to 2 mM enhanced the degree of EPSP reduction as expected (2 mM Ca^{2+}: 0.32 ± 0.04, n = 4, $p < 0.05$ for 2 mM Ca^{2+} vs. CTRL/4 mM Ca^{2+} and $p < 0.05$ for 2 mM Ca^{2+} vs. TTX/4 mM Ca^{2+}).

ii. Reducing the extracellular Ca^{2+} concentration is known to alter short-term plasticity and to facilitate successive responses following repetitive stimulation. In 2 mM Ca^{2+} we observed a facilitation of EPSPs (shown in blue, n = 9) mimicking the responses from slices treated with TTX (red traces, taken from Fig. 4). Importantly, synaptic responses recorded in TTX-treated slices were less susceptible to γ-DGG than synaptic responses recorded in 2 mM control slices even though train responses were similar indicating that the changes in short-term plasticity are indeed mediated postsynaptically.

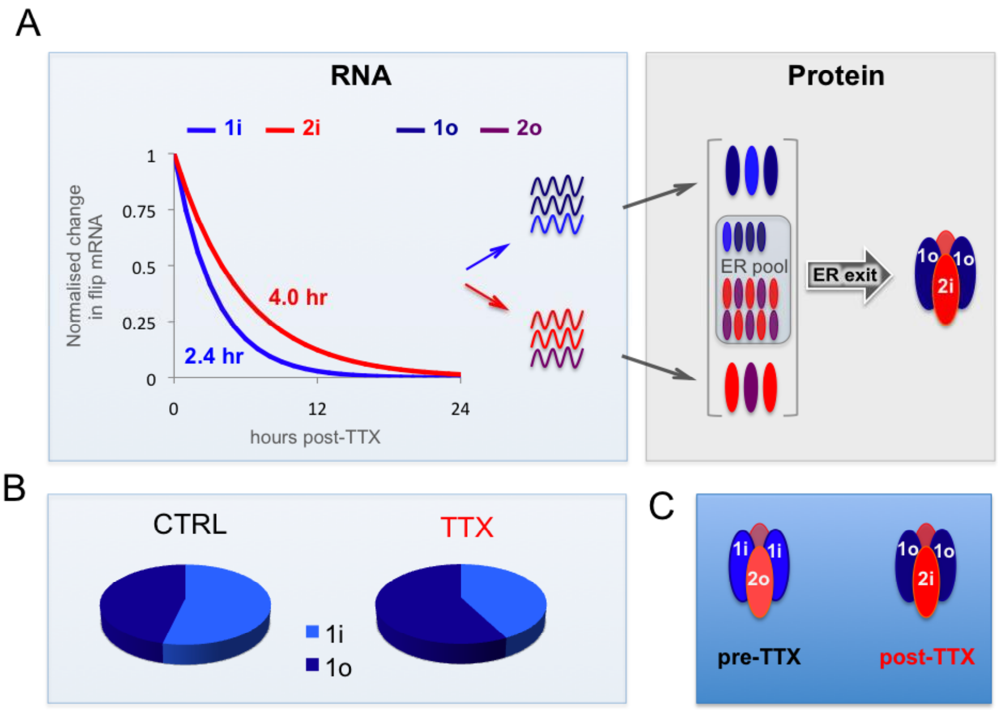

Figure S7

Figure S7 (related to Figure 1). Summary of the splicing-driven AMPAR assembly model

A. Summary schematic of how GluA1, 2 mRNA and protein turnover affects splicing-driven AMPAR remodeling. Left panel (RNA): different GluA1, 2 mRNA turnover rates result in more rapid decay of 1i mRNA (light blue), relative to 2i (red), giving rise to a speedier increase of 1o levels (dark blue wavy lines) in the initial 12 hrs post TTX. Right panel (Protein): GluA1, 2 protein half-lives in the ER differ substantially - Q/R edited GluA2 gives rise to a stable ER pool (small ellipsoids), which is not seen for GluA1 (Greger et al., 2002). As a result substantially less GluA1 protein will reside in the ER at steady state; together with the increased levels of newly made 1o mRNA substrate, 1o protein is expected to prevail after TTX (newly made protein, added to the pre-existing ER pool, is shown as larger ellipsoids). Accordingly, 1o will sample from a mixed 2i/o pool during subunit assembly giving rise to predominant assembly and forward traffic of 1o/2i AMPAR heteromers.

B. Pie charts illustrating the change in GluA1 splice variant mRNA expression in control slices and slices treated with TTX for 48 hours. The predominant GluA1 splice variant switches to flop following activity deprivation.

C. The splice variant composition of GluA1/2 heteromers changes after silencing with TTX

See also Figures 1E, S1D and E.

Table S1. AMPAR kinetics and pharmacological properties in hippocampal subfields

		CA1				CA3			
		Median	Mean	SEM	N†	Median	Mean	SEM	n†
Deactivation	τ (ms)	2.8	2.8	0.1	19 (12)	3.6	3.6	0.2	4 (2)
Desensitization	τ_{fast} (ms)	7.6	7.8	0.4	37 (22)	9.8	10.3	1.1	8 (4)
	Amp_{fast} (%)	62.6	61.8	2.1		57.8	57.2	1.4	
	τ_{slow} (ms)	28.6	29.9	1.6		36.1	37.4	2.56	
	Amp_{slow} (%)	37.4	38.2	2.1		42.2	42.8	1.4	
	$\tau_{weighted}$ (ms) *	14.2	15.9	0.8		20.7	21.8	1.3	
	% desensitization	98.0	97.6	0.3		95.9	95.0	0.9	
SS/Peak in 10 μM cyclothiazide (%)		45.9	46.8	3.0	33 (23)	76.7	76.6	3.1	8 (6)

† The sample size is the number of cells. The number of slices is indicated in brackets.

* Note the similarity of our weighted desensitization time constants with those published for somatic and dendritic patches excised from CA1 (18.5 and 18.2 ms respectively) and CA3 (23.4 and 20.1 ms respectively) pyramidal cells in acute rat hippocampal slices (Spruston et al., 1995)

Supplemental Experimental Procedures

Preparation for roller-tube slice cultures

Coverslips (12 x 24 mm, Kindler O'GmbH) were placed into alumina holders (8542E40, Thomas Scientific) and washed (with sonication) in Millipore water, 96 % ethanol and then allowed to dry on sterile tissue paper prior to autoclaving. All the following procedures were carried out under sterile conditions, dissection tools were baked and solutions were filtered. Coverslips were returned to the autoclaved holders and submerged in freshly prepared 25 μg/ml poly-D-lysine solution (30 kDa, P7280, Sigma). After allowed to coat for 5 minutes, coverslips were washed three times with autoclaved Millipore water and allowed to dry on sterile tissue paper. Collagen Type I from rat tail (27666, Fluka) was prepared in aqueous solution at 1 mg/ml and 50–100 μL was spot into the centre of each coverslip and spread to a diameter of ~10 mm. Cross-linking to form a collagen matrix was achieved by exposing coverslips to ammonia vapour from droplets of 5 N ammonium hydroxide solution (318612, Sigma) for 5–10 minutes. Coverslips were then washed in autoclaved Millipore water (containing 10 μg/ml phenol red) until further washing caused no change of indicator colour. Collagen-coated coverslips were maintained in a sealed jar containing Hanks balanced salt solution (HBSS, GIBCO) for up to a week prior to the continuing the remainder of the protocol.

Slice preparation and treatments

All procedures were carried out in accordance with UK Home Office regulations. Sprague-Dawley rat hippocampi were dissected from pups (postnatal age 5 days) in a sucrose-modified Gey's balanced salt solution, which was (in mM): sucrose (175), NaCl (50), KCl (2.5), Na_2HPO_4 (0.85), KH_2PO_4 (0.66), $NaHCO_3$ (2.7) $MgSO_4$ (0.28), $MgCl_2$ (2), $CaCl_2$ (0.5), glucose (25) and 10 μg/ml phenol red (~ 330 mOsm, pH 7.3). Transverse hippocampal slices (300–400 mm thick) were cut using a McIllwain tissue chopper and individually transferred in dissection medium using a wide-lumen pipette and positioned with a fine paint brush on the surface of the collagen matrix in the centre of the coverslip. Dissection medium was carefully aspirated and the slices were

wet with a couple of drops of pre-equilibrated (37 °C / 5 % CO_2) slice culture medium. Culture medium contained 50 % Basal Medium Eagles (BME), 25 % Hank's or Earle's balanced salt solution (HBSS or EBSS), 25 % heat-inactivated horse serum, 1 mM L-Glutamine (all from GIBCO), and 6.5 g/L D-glucose (320 mOsm). The medium did not contain antibiotics. Coverslips were transferred into flat-bottomed polystyrene culture tubes (156758, Nunc) containing 0.75 ml culture medium, the screw cap was sealed tight and the slices were maintained in the culture incubator overnight. The following day culture tubes were transferred to a custom-made roller drum: angled ~8 ° and rotating at ~10 rotations per hour (rph). Throughout the entire culture process, incubator was set to 36 °C with no humidity or CO_2 control. At 3-4 days *in vitro* (DIV), slices were fed with culture medium supplemented with 1 μM each cytosine β-D-arabinofuranoside (araC), 5-fluoro-2'-deoxyuridine (FUDR) and uridine (Sigma). Slices were fed the following day and twice weekly thereafter (without antimitotics).

Slices were cultured for at least 3 weeks prior to treatments. For treatments, slices were fed with culture medium containing TTX (1 μM), or no drug (CTRL). Slices were returned to the incubator for a duration of 48 hours, after which they were dissected for molecular biology or used for electrophysiological recordings. Slices grown via the interface method, on membrane inserts (0.4 μM Milicell, Millipore), gave quantitatively comparable results.

Molecular Biology

RNA was extracted from tissue with Trizol according to the manufacturer's instructions (Invitrogen). Total nucleic acid was treated with DNase I, and used for random primed cDNA synthesis catalyzed by M-MLV reverse transcriptase (Promega). PCR was conducted with *Taq* polymerase (Invitrogen) using standard protocols. PCR amplicons were sequenced directly (Source Bioscience Lifesciences, UK); peak heights in sequence chromatograms were quantified using PeakPicker software (Ge et al 2005). To quantify i/o splicing, the mean of the first five different base positions for the alternatively spliced exon was used.

Expression analysis

RT-PCR was conducted using standard instruments and protocols (see Experimental Procedures). The ROI in GluA1, 2, 3 were amplified from cDNA with primer pairs at 0.5 μM final concentration for 35 cycles. Type I TARPs (γ-2, 3, 4 and 8) were amplified from cDNA by nested PCR using pan-TARP primers. Amplification gives higher molecular weight band (413 bp) corresponding to γ8, and lower molecular weight bands (377 and 386 bp) for γ-2/3 and γ4 respectively. The lower molecular weight fragments were digested with restriction enzymes *XhoI*, *ClaII* and *StyI*, which have specific recognition sequences for γ-2, 3 and 4 respectively. For TARP amplification from hippocampal cDNA, γ-4 was below the level of detection as confirmed with a *StyI* digest. (Monyer and Lambolez, 1995). Cornichons 2 and 3 were amplified from cDNA by reverse transcription PCR using pan-CNIH primers. Amplification results in a 384 bp band, corresponding to both CNIH2/3. PCR products were digested with restriction enzymes *NciI* and Sca*I*, which have specific recognition sequences for CNIH2 and 3. All primer sequences are available upon request.

PCR amplicons or RFLP digest products were run on agarose gels and post-stained with ethidium bromide. Quantifications were made on gel images by measuring band peak intensities using ImageJ (NIH, http://rsb.info.nih.gov/ij/). To determine the extent of editing and mutually exclusive splicing, PCR products were cleaned up using ExoSAP-IT (USB Corporation) and sequenced.

Real-time PCR

The expression levels of GluA1 + 2, relatively to GAPDH, were measured by real-time RT-PCR using TaqMan® pre-developed assays (Applied Biosystems). Predesigned TaqMan assays (GluA1 00709588_m1, GluA2 00568514_ml and Gapdh 4352338E; ABI). Assays were used and validated according to manufacture protocols. PCR reactions were prepared in a final volume of 10 μl, containing 0.5 μl of 20x TaqMan probe mix for each gene of interest (FAM labeled) and GAPDH (VIC labeled), 4 μl diluted cDNA and 5μl of 2× TaqMan Universal PCR Master Mix (Applied Biosystems). PCR reactions were run on a Rotor-Gene 6000 (Qiagen) under

the following thermal conditions: 95°C for 10 min (to activate the polymerase), followed by 45 cycles of denaturation at 95°C for 15 s, and a combined annealing and extension step at 60°C for 45s. Additionally, expression levels of CNIH2, relatively to beta-microblobulin, were measured by Sybr-green RT-PCR. PCR reactions for each gene were prepared in a final volume of 10 µl, containing 1 µl of 5 µM primers, 5µl of 2× TaqMan Universal PCR Master Mix (Qiagene) and 4 µl diluted cDNA. PCR reactions were run on a Rotor-Gene 6000 under the following thermal conditions: 95°C for 10 min, followed by 40 cycles of denaturation at 95°C for 5 s, annealing at 60°C for 10s and extension step at 72°C for 10s. Post-reaction data were analyzed with Rotor-Gene Software 1.7. The delta-delta C_T method was used for analysis.

Electrophysiology – fast solution exchange to excised patches

Slices were transferred to a recording chamber superfused at 3 ml/min with Tyrode’s solution, which was (in mM): NaCl (137), KCl (2.5), $CaCl_2$ (2.8) $MgCl_2$ (2) $NaHCO_3$ (11.3), NaH_2PO_4 (0.6), HEPES (2 or 5, pH 7.3) and glucose (25) (osmolarity was ~310 mOsm). The solution was supplemented with 10 µg/ml phenol red to monitor pH. MK-801(+) and kynurenic acid were added to the Tyrodes perfusate at 10 nM and 0.5 – 1 mM respectively to protect the slice during agonist application to outside-out patches.

Outside-out patches were excised from pyramidal cells under visual guidance on an inverted microscope (Diaphot 200, Nikon) using infrared-differential interference contrast (IR-DIC) optics. Glass pipettes had an open-tip resistance of 2–5 MΩ and were filled with intracellular solution consisting of (in mM): CsF (120), CsCl (10), EGTA (10), $MgCl_2$ (2), Na_2-ATP (2), HEPES (10) to pH 7.3 with CsOH. For recordings of current-voltage (I/V) relationships, this was supplemented with (in mM): QX-314 Cl (1) and spermine (0.1). This concentration of added spermine gave a final free spermine concentration of between 26.8 µM (Soto et al. 2007) and 46.3 µM (Watanabe et al.1991); calculations were performed by Solver (Frontline Systems Inc) in Excel (Microsoft Corporation).

Filtered current recordings (5 kHz, Bessel) with a holding potential of -60 mV were obtained at room temperature (20–26 °C) using an Axopatch-1D amplifier and

acquired at 20 kHz sampling rate using pClamp 9 software (Molecular Devices). Glutamate (3 or 10 mM) was applied using a θ-pipette and a high-Na extracellular solution as described previously (Penn et al., 2008; Rossmann et al., 2011). High-Ca extracellular solution was (in mM): $CaCl_2$ (50), NMDG (90), glucose (10), HEPES (10) to pH 7.4 with HCl. DL-AP5 (0.05 - 0.1 mM) was routinely added to inhibit NMDA receptor currents in patches from neurons. Other drugs used depending on the experiment were (in mM): cyclothiazide (0.01), kainate (0.5), GYKI 52466 (0.01) and philanthotoxin 74 (0.1); all were from Tocris Bioscience. The latter was washed-in at -80 mV (or washed-out at +40 mV) whilst applying 1 ms pulses of L-Glu at 10 Hz for a duration of 3 minutes. Analysis was performed in Axograph 4.8 (Molecular Devices) and using custom scripts written in GNU Octave 3.2.0 (http://www.octave.org).

Current-voltage (I-V) relationships were obtained from peak currents evoked by 1 ms applications of L-Glu at holding potentials ranging from +50 to -70 mV in 10 mV inter-sweep increments separated by 2 second time intervals. Analysis of I-V data was carried out using the *fitIV* octave script to obtain the reversal potential (E_{rev}) and the rectification index (g_{+10}/g_{-40}) as described previously (Rossmann et al., 2011). The ratio of calcium permeability relative to that of sodium (P_{Ca} / P_{Na}) was calculated from the reversal potentials of glutamate-evoked currents in outside-out patches in high Ca ($E_{rev,Ca}$) and high Na ($E_{rev,Na}$) extracellular solutions using the following equation: $0.25\ \alpha_{Na}/\alpha_{Ca}\ (\exp[(2\ E_{rev,Ca} - E_{rev,Na})\ F/RT] + \exp[(E_{rev,Ca} - E_{rev,Na})\ F/RT])$, where F, R and T have their conventional meaning and α is the ion activity calculated as the product of the ion concentration and activity coefficient. The activity coefficients (γ) were estimated by interpolation of tabulated values for a pure 145 mM NaCl solution (γ_{Na} = 0.758) and for 50 mM $CaCl_2$ in a mixture with 90 mM NaCl (γ_{Ca} = 0.523) (Butler, 1968). The $E_{rev,Ca}$ and $E_{rev,Na}$ values were corrected for calculated liquid junction potentials of 13.1 and 9.2 mV respectively.

For analysis of desensitization kinetics, current recordings of prolonged glutamate application to outside out patches (10 mM for 500 ms) were leak subtracted (typically 12 pA corresponding to 5 GΩ patch seal for -60 mV holding potential) and 100 to 200 ms of L-Glu evoked current was exported for subsequent analysis in GNU Octave (http://www.octave.org) using a custom script *fitexp*, which performed the following

series of functions. Data was initially fit by linear least-squares Chebyshev algorithm coded in a custom function *chebexp*, based on (Malachowski et al., 2007). The residuals of fits with up to 4 exponents were evaluated for their adjusted R-squared and F ratios (Horn, 1987; Motulsky and Christopoulos, 2004). Desensitization was consistently fit well to two exponents, although more real exponents were sometimes achievable. The Chebyshev-derived parameters of the selected fit (2 exponents) were thus used as starting values in an iterative Nelder-Mead Simplex optimization to minimize the sum-of-squared error (SSE, octave function *fminsearch*, right-angled simplex; relative size = 1×10^{-6}), which on average gave ~ 5% improvement. The quality of the selected fit for every recording was confirmed by plotting graphs of the residuals, the fitted line overlaying the data points, and of the individual exponents. The weighted exponential time constant was calculated as the sum of component time constants multiplied by their fractional amplitude. Approximately 1/10 cells from CA1 stratum pyramidale had a weighted desensitization time constant < 10 ms, which were thus were considered interneurons and not included in subsequent analyses (Geiger et al., 1995). For analysis of responses to high-frequency bursts of L-Glu, where rapid solution exchange is critical, recordings with 20-80 % rise times > 0.4 ms or deactivation time constant > 4 ms were discarded. Under these conditions, the amplitude of the intial peak in the train was ~ 90 % of the amplitude of the response to prolonged glutamate application, and this was invariant between treatments. Effective tip positioning was routinely confirmed at the end of the experiment by blowing off the patch and checking the open tip response.

Recording of mEPSCs was conducted at room temperature with the same intracellular solution used for the recording of evoked EPSCs (see below). Tyrodes solution was supplemented with 1 μM TTX, 50 μM AP5 and 20 μM Bicuculline to isolate AMPAR-mediated mEPSCs. mEPSCs were recorded at a holding potential of -70 mV. Signals were filtered online at 2 kHz (-3 dB, Bessel) and acquired at a sampling frequency of 10 kHz. The recording was monitored with depolarizing voltage steps (-5 mV, 20 ms) from a holding potential of -70 mV. Access resistance (R_a) was calculated by dividing the voltage step with the transient current peak. No series resistance compensation was used.

Event detection, peak measuring and alignment were carried out semi-automatically using custom scripts written in GNU Octave (http://www.octave.org): *events*, *peaker* and *avgtraces*. Briefly, candidate events were identified from filtered traces (High-pass: 2 Hz; Low-pass: 0.91 kHz; -6 dB cut-off, Binomial) using a level detection threshold of 4 pA for ≥1 ms. Candidate events were then extracted from the *unfiltered* data for automatic peak/baseline detection and rise-time measurement. Individual mEPSCs were considered those where the ~40 ms post-peak period could be fit using a Chebyshev algorithm with a single exponential. Individual mEPSCs accounted for ~80% of candidate events and a total of 50 individual mEPSCs were collected per cell. The median amplitude, 20-80 % rise-time and decay time constant of individual mEPSCs was calculated per cell. The mEPSC frequency per cell was calculated as the reciprocal of the median time interval between individual mEPSCs. Mean mEPSCs were obtained for each cell following alignment of individual mEPSCs to the rising slope (20 % rise-time point). The mean (± SEM) of the mEPSC statistics were calculated for cells within each treatment (CTRL and TTX).

Details of HEK 293T cell transfection and electrophysiology have been described previously (Rossmann et al., 2011). To assay heteromerization, the transfection ratio of GluA1 and GluA2 was always 4:1 and rectification index (RI) calculated as the ratio of slope conductance (*g*) at +10 mV over -40 mV (relative to E_{rev}). For characterizing properties of GluA1/2 heteromers this transfection ratio was varied (ranging from 1:1 to 1:9) to achieve optimal expression of GluA1/2 heteromers and only patches with RI > 0.7 were included in the analysis.

Recordings of evoked synaptic AMPAR EPSPs

Hippocampal organotypic slice cultures were transferred to a submerged recording chamber and continuously perfused with ACSF (mM): NaCl 125, $NaHCO_3$ 25, KCl 3, NaH_2PO_4 1.25, $CaCl_2$ 2, $MgCl_2$ 1, sodium pyruvate 3, and glucose 25, oxygenated at 95% O2 and 5% CO2. Whole-cell recordings from CA1 neurons were performed at room temperature or at 34-37°C (to speed up kinetics to enable recordings at higher stimulation frequencies), with a current clamp amplifier (BVC 700A, Dagan). Borosilicate glass capillaries with a resistance of 3-5 MΩ were filled with a solution

containing (mM): potassium gluconate 135, NaCl 7, HEPES 10, Na_2-ATP 2, Na-GTP 0.3, $MgCl_2$ 2, and with Alexa Fluor 568 (0.01-0.04, Molecular Probes) for visualization after recording (pH was adjusted to 7.2–7.3 with KOH). CA1 pyramidal cell identity was first confirmed by the presence of spike frequency adaptation in response to a depolarizing current injection and the presence of a sag potential in response to a hyperpolarizing current injection. Synaptic excitatory postsynaptic potentials (EPSPs) were evoked by Schaffer collateral/commissural fiber stimulation (0.33 Hz). AMPAR-mediated EPSPs were recorded in the presence of D-APV (50 µM), SR 95531 (2 µM) and CGP 52432 (2 µM) at a membrane potential of ~ -80 mV. Calcium and magnesium concentrations were raised to 4 mM to dampen excitability. NMDAR-mediated EPSPs were elicited in the presence of NBQX (10 µM), SR 95531 and CGP 52432 at normal resting membrane potentials of ~ -60 mV. To investigate changes in STP afferent fibers were stimulated repetitively with five pulses delivered at 10 (at room temperature, ~22˚C) or 50 Hz (near physiological temperature, 37˚C). Signals were low-pass filtered at 10 kHz and acquired at 25–40 kHz using an ITC-18 interface (HEKA) controlled by an Apple computer. Data were analyzed using Axograph X (Axograph Scientific) and Excel (Microsoft).

Neuronal reconstructions

A fraction of CA1 neurons were filled with biocytin (0.5%, added to the pipette recording solution); slices were then fixed overnight in 4% PFA/PBS, pH 7.4. Slices were next incubated for 12–24 h in PBS supplemented with 0.5–2% Triton X-100 and 0.2% streptavidin, Alexa Fluor 488 or Alexa Fluor 568 conjugate (Invitrogen) at 4–6 °C. Images were aquired on a Zeiss 710 confocal microscope.

Data analysis

Most statistical comparisons were performed using in Prism or Instat (Graphpad Software Inc). Other statistical analyses were performed using R 2.81 (http://www.rproject.org) or GNU Octave (http://www.octave.org). All statistical tests were two tailed using a significance level of 0.05. In cases were distributions deviated significantly from normality, or where variances were unequal, non-parametric

methods were used. Graphical presentation of data was carried out in Excel (Microsoft Corporation) or Prism (Graphpad Software Inc).

Supplemental Text

To analyse subfield alternative mRNA processing, we analysed RNA expression from whole subfield extracts and interpreted the data as being representative of pyramidal cell expression profile since CA1 pyramidal neurons are expected to prevail (~90%) (Olbrich and Braak, 1995). It has been shown that astroglia are largely localized to the dendritic layers in CA1, stratum oriens and s. radiatum (Nixdorf-Bergweiler et al., 1994), and even there, cell processes from glia (not exclusively astroglia) occupy less than a tenth of the tissue volume (Mishchenko et al 2010). In contrast, astroglia are much less frequent in s. pyramidale (Nixdorf- Bergweiler et al., 1994), which we have analyzed selectively; in fact our stratum pyramidale preparation RNA splicing profiles closely resemble data for total CA1. Therefore, a major contamination from astroglia does not seem likely. Furthermore, when comparing CA1 mRNA profiles from freshly cut (P5) slices with the RNA pattern obtained 21 days later (21 days in vitro) we observe a trend towards elevated flop expression (unpublished observations, A.B. and I.H.G.) This is consistent with developmental profiles of splice forms reported by *in situ* hybridization for cells in s. pyramidale (Monyer et al., 1991), but opposite to the developmental changes reported for astroglial cells (Seifert et al. 1997, 2003). An enrichment of astroglial cells specifically in our culture preparation is unlikely since an antimitotic treatment was used, which will reduce growth of astrocytes. Taken together, these points make a major contribution of astroglial mRNA unlikely.

Rectification of AMPAR currents was used to assay heteromerization competence of recombinant subunits in the presence or absence of TARPs (Figures 2C, S4B). ATP, which was included in the patch pipette solution, binds spermine, which could potentially limit its use for distinguishing between Ca2+-permeable and -impermeable AMPARs. This is especially true in the presence of TARPs, which lower efficacy of spermine binding (Soto et al., 2007). In our intracellular solution (containing 2 mM ATP, 2 mM Mg^{2+}) by the same calculation we have a free spermine concentration of 26.8 μM, which is well above the Kd(0) (the apparent dissociation constant at 0 mV)

of spermine for AMPARs in the absence or presence of TARPs (0.49 and 4.54 μM respectively, Soto et al, 2007). Relating to this, we note that in our index for rectification, we use slope conductance at the modestly positive +10 mV and therefore at this driving force consider that spermine permeation through the pore (which would violate the model from which the Kd(0) was calculated) to be negligible. Therefore, under our experimental conditions we consider that our rectification index is a suitable assessment of AMPAR composition, even in the presence of TARPs. Moreover, we could confirm the efficacy of our free spermine concentration to block outward currents of A1 homomers in the presence of stargazin (γ-2).

The splice variant composition of native A1/A2 heteromer was characterized using cyclothiazide: a drug that binds at the LBD dimer interface and shows greatest affinity for flip variants, and more so for the A2 subunit. The splice variant selectivity of cyclothiazide is reportedly occluded by the presence of stargazin (Tomita et al. 2006). Stargazin/γ-2 coexpression with AMPARs boosts cyclothiazide potentiation, and it does so more for A1o than for A1i. However, even with a 1:1 coexpression of A1 with γ-2 there is still a 2-fold difference between the splice variants (Tomita et al. 2006). We confirmed this also by measuring CTZ efficacy of recombinant A1i/A2o and A1o/2i combinations co-transfected with γ-8 (Figure 2B). This demonstrates that even in the presence of relevant TARPs, that A2i (rather than A1i) dominates CTZ efficacy in A1/A2 heteromers.

Our results also indicate that 2i dominates desensitization/resensitization kinetics of heteromeric receptors. We point out this only appears to hold true for the A1/2 heteromers. For example, recombinant A2i/A3o receptors do not show a greater effect on desensitization than A2o/A3i (Mosbacher et al 1994). Furthermore, the time constant of the A2i-containing heteromer is only increased to greater than that of the A2-lacking homomer for A1, and not for A3 or A4 homomers (Mosbacher et al 1994). Therefore, the kinetic dominance of A2i is exclusively for the widely expressed A1/A2 combination. We confirmed that this exists also for recombinant A1/A2 heteromers in the presence of either γ-2 or γ-8 (Figure S4D)

Supplemental References

Balik, A., Penn, A.C., Nemoda, Z., and Greger, I.H. (2012). Activity-regulated RNA editing in select neuronal subfields in hippocampus. Nucleic Acids Res. *in press*.

Butler, J.N. (1968). The thermodynamic activity of calcium ion in sodium chloride-calcium chloride electrolytes. Biophys J *8*, 1426-1433.

Geiger, J.R., Melcher, T., Koh, D.S., Sakmann, B., Seeburg, P.H., Jonas, P., and Monyer, H. (1995). Relative abundance of subunit mRNAs determines gating and Ca2+ permeability of AMPA receptors in principal neurons and interneurons in rat CNS. Neuron *15*, 193-204.

Greger, I.H., Khatri, L., and Ziff, E.B. (2002). RNA editing at Arg607 controls AMPA receptor exit from the endoplasmic reticulum. Neuron *34*, 759-772.

Hessler, N.A., Shirke, A.M., and Malinow, R. (1993). The probability of transmitter release at a mammalian central synapse. Nature *366*, 569-572.

Horn, R. (1987). Statistical methods for model discrimination. Applications to gating kinetics and permeation of the acetylcholine receptor channel. Biophys J *51*, 255-263.

Lei, S., and McBain, C.J. (2004). Two Loci of expression for long-term depression at hippocampal mossy fiber-interneuron synapses. J Neurosci *24*, 2112-2121.

Malachowski, G.C., Clegg, R.M., and Redford, G.I. (2007). Analytic solutions to modelling exponential and harmonic functions using Chebyshev polynomials: fitting frequency-domain lifetime images with photobleaching. J Microsc *228*, 282-295.

Monyer, H., and Lambolez, B. (1995). Molecular biology and physiology at the single-cell level. Curr Opin Neurobiol *5*, 382-387.

Motulsky, H.J., and Christopoulos, A. (2004). Fitting Models to Biological Data using Linear and Nonlinear Regression (New York: Oxford University Pres, Inc).

Penn, A.C., Williams, S.R., and Greger, I.H. (2008). Gating motions underlie AMPA receptor secretion from the endoplasmic reticulum. EMBO J *27*, 3056-3068.

Rosenmund, C., Clements, J.D., and Westbrook, G.L. (1993). Nonuniform probability of glutamate release at a hippocampal synapse. Science *262*, 754-757.

Rossmann, M., Sukumaran, M., Penn, A.C., Veprintsev, D.B., Babu, M.M., and Greger, I.H. (2011). Subunit-selective N-terminal domain associations organize the formation of AMPA receptor heteromers. EMBO J *30*, 959-971.

Shen, Y., Hansel, C., and Linden, D.J. (2002). Glutamate release during LTD at cerebellar climbing fiber-Purkinje cell synapses. Nat Neurosci *5*, 725-726.

Spruston, N., Jonas, P., and Sakmann, B. (1995). Dendritic glutamate receptor channels in rat hippocampal CA3 and CA1 pyramidal neurons. J Physiol *482 (Pt 2)*, 325-352.

Wadiche, J.I., and Jahr, C.E. (2001). Multivesicular release at climbing fiber-Purkinje cell synapses. Neuron *32*, 301-313.
